# Comparison of creatinine-based equations for estimating glomerular filtration rate in deceased donor renal transplant recipients

**DOI:** 10.1371/journal.pone.0231873

**Published:** 2020-04-28

**Authors:** Luciano da Silva Selistre, Sandrine Lemoine, Allyriane Dantec, Fanny Buron, Vandréa Carla de Souza, Mariana Bertoldo, Carlos Eduardo Poli-de-Figueiredo, Thomas Rimmelé, Olivier Thaunat, Lionel Badet, Emmanuel Morelon, Antoine Sicard, Laurence Dubourg

**Affiliations:** 1 Néphrologie, Dialyse, Hypertension et Exploration Fonctionnelle Rénale, Groupement Hospitalier Edouard Herriot, Hospices Civils de Lyon, Lyon, France; 2 Universidade de Caxias do Sul—Programa de Pós-Graduação em Ciências da Saúde, Caxias do Sul, Brazil; 3 Hospital Geral de Caxias do Sul, Caxias do Sul, Brazil; 4 Université Lyon 1, Lyon, France; 5 CarMeN: Cardiovasculaire, Métabolisme, Diabétologie & Nutrition-INSERM U1060/Lyon 1, Lyon, France; 6 Service de Transplantation, Néphrologie et Immunologie Clinique, Hôpital Edouard Herriot, Lyon, France; 7 Pontifícia Universidade Católica do Rio Grande do Sul, Porto Alegre, Brazil; 8 Service d'anesthésie-réanimation, hôpital Edouard-Herriot, Lyon, France; 9 Unité INSERM U1111, Lyon, France; 10 Service d’Urologie et Transplantation, Hôpital Edouard Herriot, Lyon, France; 11 Laboratoire de Biologie Tissulaire et d’ingénierie Thérapeutique (LBTI), UMR 5305 CNRS, Université Claude Bernard Lyon 1, Lyon, France; Istituto Di Ricerche Farmacologiche Mario Negri, ITALY

## Abstract

**Background:**

Estimating glomerular filtration rate (GFR) is important for clinical management in kidney transplantation recipients (KTR). However, very few studies have evaluated the performance of the new GFR estimating equations (Lund-Malmö Revised–LMR, and Full Age Spectrum–FAS) in KTR.

**Methods:**

GFR was estimated (eGFR) using CKD-EPI, MDRD, LMR, and FAS equations and compared to GFR measurement (mGFR) by reference methods (inuline urinary and iohexol plasma clearance) in 395 deceased-donor KTR without corticosteroids. The equations performance was assessed using bias (mean difference of eGFR and mGFR), precision (standard deviation of the difference), accuracy (concordance correlation coefficient—CCC), and agreements (total deviation index—TDI). The area under receiver operating characteristic curves (ROC) and the likelihood ratio for a positive result were calculated.

**Results:**

In the total population, the performance of the CKD-EPI, MDRD and FAS equations was significantly lower than the LMR equation regarding the mean [95%CI] difference in bias (-2.0 [-4.0; -1.5] versus 9.0 [7.5; 10.0], 5.0 [3.5; 6.0] and 10.0 [8.5; 11.0] mL/min/1.73m^2^, P<0.005) and TDI (17.10 [16.41; 17.88], 25.91 [24.66; 27.16], 21.23 [19.48; 23.13] and 25.84 [24.16; 27.57], respectively). Concerning the CCC, all equation had poor agreement (<0.800) without statically difference between them. However, all equations had excellent area under the ROC curve (>0.900), and LMR equation had the best ability to correctly predict KTR with mGFR<45 mL/min/1.73 m^2^ (positive likelihood ratio: 8.87 [5.79; 13.52]).

**Conclusion:**

Among a referral group of subjects KTR, LMR equation had the best mean bias and TDI, but with no significant superiority in other agreement tools. Caveat is required in the use and interpretation of PCr-based equations in this specific population.

## 1 Introduction

Accurate assessment of glomerular filtration rate (GFR) is important for the management of kidney transplant recipients (KTR).[1, 2] In addition, lower GFR at 1 year after KTR is associated with shorter allograft and patient survival [3–5] and 1-year post-KTR GFR is used as a prognosis factor.[5–7] Clinical practice guidelines therefore recommend monitoring kidney function to detect nephrotoxicity of immunosuppressive medications in order to identify early signs of rejection, to adjust drug dosage, and to estimate prognosis.[2, 6]

Ideally, GFR is measured (mGFR) with an exogenous marker (inuline, iohexol, iothalamate, Cr EDTA *etc*.).[8] However, for technical reasons GFR is most often estimated (eGFR) using equations based on plama creatinine (PCr).(1, 2) Even though the performance of PCr equations in chronic kidney disease (CKD) has been demonstrated, the best equation to estimate GFR after KTR is debated.[7, 9, 10] Indeed, the specific characteristics of KTR patients (immunosuppressive treatments, history of chronic kidney disease (CKD),decreased muscle mass *etc*.) can change the performance of PCr-based equations estimating GFR established in CKD patients.[7, 11] Furthermore, the majority of 1-year post-KTR patients have a GFR below 60 mL/min/1.73m^2^.[9, 11–13] Most of the time, GFR is estimated using the Chronic Kidney Disease–Epidemiology Collaboration (CKD-EPI) or the Modification of Diet in Renal Disease Study (MDRD).[3, 5, 11, 12, 14–16] Recently, new PCr-based equations have been proposed to calculate eGFR in the general population, such as Lund-Malmö Revised (LMR)[17] and Full Age Spectrum (FAS),[18] but their performance in the KTR population has yet to be evaluated.

The present study was conducted to assess the performance of the most commonly used PCr-based equations (CKD-EPI and MDRD) and the most recently published PCr-based equations (LMR and FAS) in a cohort of deceased-kidney-transplant recipient 1-year after graft.

## 2 Materials and methods

### 2.1 Study population

**Fig 1:**
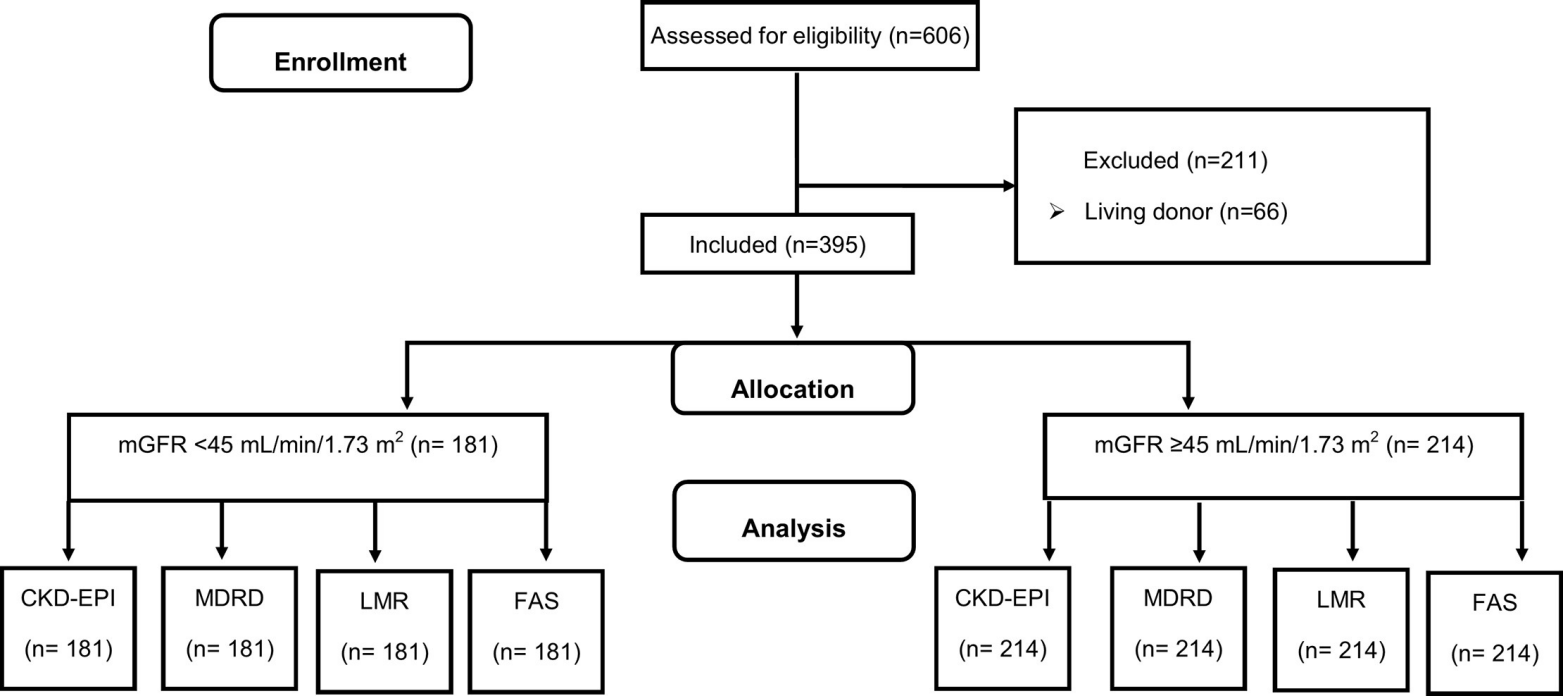
Flow chart of the study. GFR, glomerular filtration rate; CKD-EPI, chronic kidney disease epidemiology collaboration equation; MDRD, Modification of Diet in Renal Disease Study; LMR, Lund-Malmö revised equation; FAS: full age spectrum equation.

The study considered a cross-sectional retrospective sample of 395 patients with KTR from deceased donors in a regional center of Transplant (Clinical Immunology and **Transplantation** department, Edouard Herriot Hospital, Lyon, France). All patients were adults (≥ 18 years old) referred to undergo a routine GFR measurement one year after transplantation between June 2009 and June 2015. At that time the immunosuppression consisted of either tacrolimus in combination with mycophenolate sodium. Tacrolimus was C_0_ monitored with a therapeutic window of 5 to 10 μg/L and mycophenolate sodium ≥1.9 mg/L. The exclusion criteria were treatment by living donor, multiple transplantation (*e*.*g*. pancreas, liver), corticoid, cyclosporine A and trimethoprim treatments (Fig 1). All procedures were carried out in accordance with the ethical standards of the institutional and/or national research committee and with the 2013 Helsinki Declaration and its later amendments or with comparable ethical standards. Precisely, an appropriate informed consent was obtained from each participant or his/her legal representatives. The consent form included information on the procedure itself as well as on the possibility of later use of the data for research purposes. According to French law applicable at the time of the study, an observational study that did not change routine management of patients did not need to be declared or submitted to a research ethics board (Loi Huriet-Sérusclat 88–1138, 20 December 1988 and its subsequent amendments, text available at http://www.chu-toulouse.fr/IMG/pdf/loihuriet.pdf). None of the transplant donors was from a vulnerable population and all donors or next of kin provided written informed consent that was freely given.

Reliability assessment and comparisons between the four eGFR equations (CKD-EPI, MDRD, LMR, and FAS) were carried out on different subgroup of mGFR levels: <45 and ≥45 mL/min/1.73m^2^.

### 2.2 Laboratory assessments

#### 2.2.1 Measured GFR assessment

The mGFR was performed using standard methods (urinary inulin or plama iohexol clearance). Briefly, urinary inulin clearance (GFRin) (Inutest 25%; Fresenius, Kabi, Austria) was performed with a continuous infusion of polyfructosan 40 mg/kg after a priming dose of 30 mg/kg. Water diuresis was induced by oral administration of 5 mL/kg of water followed by 3 mL/kg every 30 min combined with an intravenous infusion of 0.9% sodium chloride. This enabled the patients to spontaneously empty their bladder every 30 min. Three to four urine samples were collected, and a blood sample was drawn mid-way through each collection period. The clearance value, calculated by the usual UV/P formula, was the mean value of three to four clearance periods. Plasma and urine polyfructosan were measured using the same enzymatic method, which has demonstrated very good specificity and reproducibility (within-run precision <1% and between-run precision <3.5%).[19]

Iohexol plasma clearance (GFRio) was performed according to a standard technique that used a single-bolus injection. An IV injection of 6 mL of iohexol (Omnipaque 300 mg/mL; GE Healthcare SAS, Vélizy-Villacoublay, France) was administered, and blood samples were drawn from the contra lateral arm after 120, 180, and 240 min. The GFRio was calculated from the slope of plasma concentrations using a 1-compartment model corrected using the Bröchner-Mortensen formula. The results were expressed per 1.73 m^2^ according to the Dubois formula. The serum iohexol concentration was measured by High Performance Liquid Chromatography (HPLC). External quality control was provided by Equalis (Uppsala, Sweden) every 3 months.

In our service with a sample of 140 non-KTR patients (unpublished data), the adjustment equation by Passing-Bablok regression [20] to GFRio versus GFRin was: GFRio = 0.92 x GFRin + 4.50. The concordance correlation coefficient was 0.970 [IC 95%: 0.960; 0.980]. Therefore, we assume that the 2 techniques are similar.

#### 2.2.2 Plasma creatinine measurement

All PCr measurements were performed using enzymatic method traceable to the National Institute of Standards and Technology (IDMS, isotope-dilution mass spectrometry-calibrated). PCr is expressed in mg/dL.

### 2.3 Estimating GFR equations

All equations that are based on PCr-IDMS standardized methods were considered in the present study (Table 1).[17, 18, 21, 22]

**Table 1:** Equations used to estimate glomerular filtration rate (mL/min/1.73m^2^).

Name	GFR estimating equations (eGFR in mL/min/1.73 m^2^)
CKD-EPI*	$Female;\;PCr\leq 0.7,eGFR=144x{\left[\frac{PCr}{0.7}\right]}^{-0.329}x\;{\left[0.993\right]}^{Age}$
	$Female;\;PCr>0.7,eGFR=144x{\left[\frac{PCr}{0.7}\right]}^{-1.209}x\;{\left[0.993\right]}^{Age}$
	$Male;\;PCr\leq 0.9,eGFR=141\;x{\left[\frac{PCr}{0.9}\right]}^{-0.411}x\;{\left[0.993\right]}^{Age}x[1.159\;if\;black*]$
	$Male;\;PCr>0.9,eGFR=141\;x{\left[\frac{PCr}{0.9}\right]}^{-1.209}x\;{\left[0.993\right]}^{Age}x\;[1.159\;if\;black*]$
MDRD*	eGFR = 175 x (PCr)^−1.154^x age^−0.203^ x [0.742 if female] x [1.159 if black *]
**LMR**	eGFR = e^x−0.0158×Age+0.438×ln (Age)^
	Female & *PCr* < 1.7: *X* = 2.50 + 0.0121 × (1.7−*PCr*)
	$Female\&PCr\geq 1.7:X=2.50–0.926\times ln\;(\frac{PCr}{1.7})$
	Male & *PCr* < 2.0: *X* = 2.56 + 0.00968 × (2.0−*PCr*)
	$Male\&PCr\geq 2.0:X=2.56–0.926\times ln\;(\frac{PCr}{2.0})$
**FAS**	$2\leq Age\leq 40:\;eGFR=107.3\;x\frac{Q}{PCr}$
	$Age>40:eGFR=107.3\;x\frac{Q}{PCr}x\;0.988^{Age-40}$
	with Q = 0.9 mg/dL in Male and 0.7 mg/dL in Female

Abbreviations: PCr: Plasma Creatinine; CKD-EPI: Chronic Kidney Disease–Epidemiology Collaboration; MDRD: Modification of Diet in Renal Disease Study; LMR: Lund-Malmö Revised; FAS: Full Age Spectrum.

* according to French recommendation the correction coefficient in Black people should not be used in European population[19]. To convert creatinine values to μmol/L, multiply by 88.4.

### 2.4 Statistical analyses

Bias (mean difference between eGFR and mGFR), precision (as the standard deviation [SD]) were by Deming Regression analysis. The accuracy was assessed using the root mean square error (RMSE) and the percentage of estimates within ±30% of the mGFR (P_30_).

The Total Deviation Index (TDI), Bland-Altman analysis and the Concordance Correlation Coefficient (CCC) were used to assess agreement between each eGFR and mGFR. The total deviation index (TDI) is a measure that captures a large proportion of data within a boundary for allowed observer’s differences. The empirical TDI was calculated for a theoretical TDI of 10% and a coverage probability of 90%. For TDI, small values (nearing zero) imply high agreement. The ideal situation would be a TDI of <10%, meaning that 90% of eGFR values fall within ±10% of mGFR, a much smaller margin of error.[9, 23] The relationship between mGFR and eGFR were illustrated using a Deming regression and the Bland-Altman scatterplot (difference mean eGFR—mGFR versus mGFR) with regression lines to limits of agreement (2.5%; 97.5% LoAs). The CCC quantifies the agreement ranging that combines meaningful components of accuracy and precision from -1 to 1, with perfect agreement at 1. CCC has the following classification according to strength of agreement: >0.990 almost perfect, 0.950–0.990 substantial, 0.900–0.949 moderate, and <0.900 poor. [9, 23] We used logarithmic transformation by CCC because the heterogeneity of the difference increased with mGFR value.

The area under the receiver-operating curve (AUC) with logistic regression was used to determine the ability of the equations estimating GFR to discriminate between patients with mGFR <45mL/min/1.73 m^2^. We used the likelihood ratio for a positive result as supplementary analysis of receiver-operating curve (ROC). The likelihood ratio for a positive result (PLR) is = sensitivity/ (1-specificity). A PLR >10.0 indicates that the test result has a large effect; PLR 5.0–10.0 indicates that the test has a moderate effect and PLR <5.0 indicates a small effect on the probability of detect disease.

The 95% confidence intervals (CIs) were calculated using the bootstrap method BCa (2,000 bootstraps). P_30_ values were compared using Cochran Q with pairwise McNemar test. AUCs were compared using bootstrapping method.

The *Holm-Bonferroni* method was used to correct for multiple comparisons and strongly controls the family-wise error rate at level alpha. The nominal *p*-value used to conclude to a statistical significance was <0.005 to bias, P_30_ and ROC area.

The analyses were performed with R for Windows, version 3.4.4 (R-Cran project, http://cran.r-project.org/).

## 3 Results

### 3.1 Characteristics of the study population

In the 395 KTR, the mean (SD) age was 52.4 (13.8) years and 39.6% of participants were women. The GFRio was used in 328 (79.2%) of the GFR measurements and mean of (SD) mGFR was 48.0 (15.1) mL/min/1.73 m^2^; and ranged from 13 to 108 mL/min/1.73 m^2^. Among all KTR 76.5% had 3 or more HLA mismatches with 120 (30.5%) had one or several acute rejection episodes (Table 2).

**Table 2:** Characteristics of the kidney transplanted recipients.

Characteristics	Total cohort	mGFR <45	mGFR ≥45	P value
Number of participants (%)	395 (100.0)	181 (46.0)	214 (54.0)	0.1
Mean (SD) age of recipient, years	52.4 (13.8)	56.7 (13.1)	48.8 (13.3)	<0.001
>60 y, n (%)	140 (35.4)	86 (47.5)	54 (25.2)	<0.001
Female sex, n (%)	164 (39.6)	74 (41.0)	82 (38.3)	0.1
Mean (SD) weight, Kg	70.2 (13.5)	70.4 (13.6)	70.0 (14.4)	1.0
Mean (SD) height, cm	167.0 (9.5)	166.5 (9.5)	167.6 (9.6)	0.3
Mean (SD) BSA, m^2^	1.80 (0.20)	1.79 (0.19)	1.78 (0.21)	0.5
Mean (SD) BMI, Kg/m^2^	25.0 (4.4)	25.3 (4.5)	24.8 (4.4)	0.9
BMI ≥30.0, n (%)	49 (12.4)	24 (13.3)	25 (11.7)	0.2
Median [IQR] PCr, mg/dL	1,43 [1,06; 1,66]	1,67 [1,40; 2,07]	1,13 [0,96; 1,33]	<0.001
Mean (SD) mGFR, mL/min/1.73 m^2^	48.0 (14.2)	36.0 (7.0)	59.0 (10.6)	<0.001
Iohexol clearance, n (%)	312 (79.0)	150 (77.3)	172 (80.3)	0.5
Median [IQR] albuminuria, mg/g	38 [17; 99]	57 [22; 151]	24 [14; 57]	<0.001
Albuminuria, n (%)				
UACR <30 mg/g	359 (92.0)	156 (86.2)	275 (94.5)	<0.001
UACR 30–300 mg/g	36 (8.0)	25 (13.8)	11 (5.5)	<0.001
CKD etiology, n (%)				
Glomerulonephritis	105 (26.5)	48 (26.5)	57 (26.5)	0.2
Hypertension	68 (17.0)	35 (19.5)	33 (15.5)	0.2
Cystic kidney disease	57 (14.5)	28 (15.5)	29 (13.5)	0.9
Interstitial nephritis	47 (12.0)	16 (9.0)	31 (14.5)	0.7
Diabetes	43 (11.0)	21 (11.5)	22 (10.5)	0.2
Indeterminate	75 (19.0)	33 (18.0)	42 (19.5)	0.2
Banff 2011 classification for acute rejection, n (%)	120 (30.5)	69 (38.0)	51 (24.0)	<0.01
Mean (SD) age of donor, years	50.2 (16.5)	56.7 (14.5)	44.6 (16.0)	<0.001
Median [IQR] PCr Donor, mg/dL	76 [57; 109]	77 [58; 109]	76 [55; 109]	0.2
HLA mismatch, n (%)				
None	5 (1.0)	2 (1.0)	3 (1.5)	0.7
One	35 (9.0)	20 (11.0)	15 (7.0)	0.4
Two	53 (13.5)	22 (12.0)	31 (14.5)	0.3
Three	118 (30.0)	54 (30.0)	64 (30.0)	0.6
Four	133 (33.5)	56 (31.0)	77 (36.0)	0.1
Five or more	51 (13.0)	27 (15.0)	24 (11.0)	0.8

mGFR, measured glomerular filtration rate in mL/min/1.73m^2^; SD, standard deviation; IQR, interquartile range; UACR, urinary albumin-creatinine ratio; HLA, Human leukocyte antigen. To convert creatinine values to μmol/L, multiply by 88.4; to convert UACR values to mg/mmol divide by 10.

### 3.2 Performance according to equation

**Fig 2:**
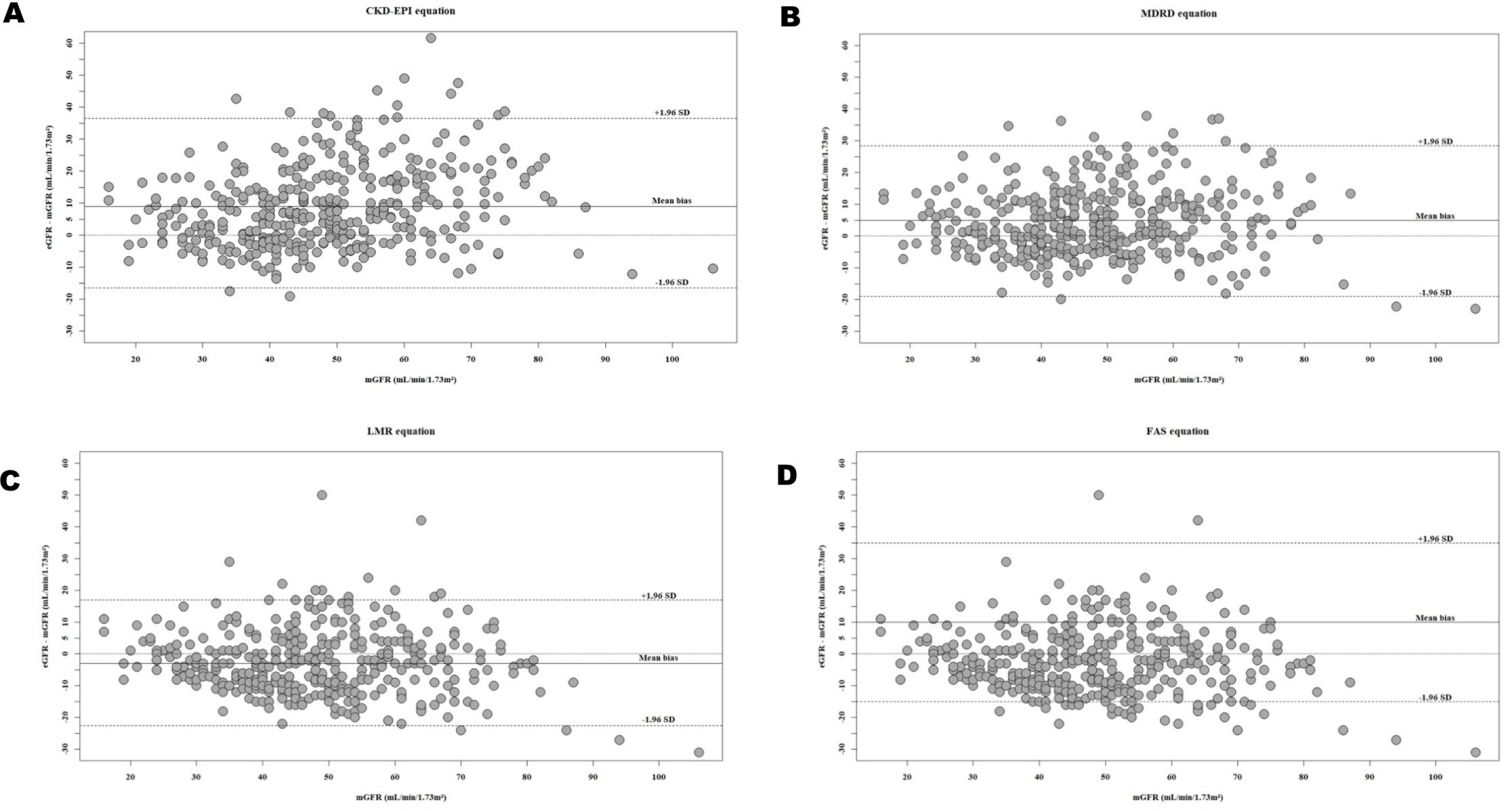
Bland and Altman plots showing the mean bias estimated GFR—measured GFR versus the measured GFR as reference standard in the cohort, using the different equations: CKD-EPI (A), MDRD (B), LMR (C) and FAS (D).

In the total cohort, the LMR equation performed better than CKD-EPI, MDRD, and FAS equations regarding the mean bias [95%CI] (-2.0 [-4.0; -1.5] versus 9.0 [7.5; 10.0], 5.0 [3.5; 6.0], and 10.0 [8.5; 11.0] mL/min/1.73 m^2^). The MDRD, CKD-EPI, and FAS equations overestimated the mGFR, whereas the LMR equation underestimated mGFR in the total population and subgroups (Fig 2 and Table 3).

**Table 3:** Bias, precision, and accuracy of the four equations estimating GFR.

	CKD-EPI equation	MDRD equation	LMR equation	FAS equation
**Total population (n = 395)**				
Mean bias, mL/min/1.73 m^2^	9.0 [7.5; 10.0]^‡^	5.0 [3.5.0; 6.0]^‡^	-2.0 [-4.0; -1.5]	10.0 [8.5; 11.0]^‡^
SD	13.0 [11.7; 14.3]	12.0 [10.0; 14.1]	10.0 [9.2; 11.5]	12.8 [11.6; 16.2]
2.5%; 97.5% LoA, mL/min/1.73 m^2^	-16.5; 36.5	-19.0; 28.5	-22.0; 17.0	-15.0; 35.0
P_30_, %	70.0 [65.5; 75.0]^‡^	81.0 [77.0; 85.0]^‡^	85.5 [82.0; 89.0]	70.5 [65.5; 74.0]^‡^
Intercept	17.5 [15.0; 20.0]	18.0 [14.5; 22.5]	17.0 [14.5; 20.0]	16.5 [13.0; 21.5]
Slope	0.54 [0.49; 0.58]	0.58 [0.48; 0.65]	0.55 [0.45; 0.62]	0.54 [0.45; 0.62]
RMSE	0.175 [0.161; 0.193]	0.179 [0.165; 0.197]	0.179 [0.165; 0.197]	0.179 [0.165; 0.198]
TDI	25.91 [24.66; 27.16]	21.23 [19.48; 23.13]	17.10 [16.41; 17.88]	25.84 [24.16; 27.57]
CCC	0.746 [0.705; 0.782]	0.785 [0.748; 0.818]	0.780 [0.741; 0.813]	0.718 [0.674; 0.757]
**mGFR <45mL/min/1.73m^2^ (n = 181)**				
Mean bias, mL/min/1.73 m^2^	4.5 [3.0; 6.0]^‡^	2.5 [1.0; 4.0]	-2.0 [-3.0; -0.5]	7.0 [6.0; 8.5]^‡^
SD	10.2 [9.0; 11.5]	6.5 [5.6; 7.8]	8.0 [7.3; 9.1]	9.4 [8.6; 10.5]
2.5%; 97.5% LoA, mL/min/1.73 m^2^	-15.0; 25.0	-15.5; 21.0	-19.0; 13.0	-11.5; 25.0
P_30_, %	70.5 [63.0; 76.0]^‡^	79.0 [72.0; 83.5]	82.0 [75.0; 87.0]	66.5 [59.0; 72.5]^‡^
Intercept	22.5 [19.5; 26.0]	22.0 [19.0; 25.5]	22.5 [19.5; 26.0]	21.0 [17.5; 24.0]
Slope	0.33 [0.27; 0.40]	0.37 [0.29; 0.45]	0.42 [0.31; 0.51]	0.36 [0.29; 0.40]
RMSE	0.173 [0.152; 0.203]	0.177 [0.155; 0.206]	0.177 [0.157; 0.208]	0.177 [0.155; 0.204]
TDI	18.55 [16.20; 21.09]	15.80 [13.77; 17.95]	14.20 [12.91; 15.61]	19.30 [17.20; 21.44]
CCC	0.550 [0.439; 0.629]	0.577 [0.449; 0.658]	0.535 [0.438; 0.620]	0.499 [0.406; 0.583]
**mGFR ≥45 mL/min/1.73m^2^ (n = 214)**				
Mean bias mL/min/1.73 m^2^	12.5 [10.0; 14.0]^‡^	6.5 [4.5; 8.0]^‡^	-3.0 [-4.0; -1.0]	12.5 [10.5; 15.0]^‡^
SD	14.2 [12.5; 16.0]	11.2 [8.4; 16.2]	11.6 [10.4; 13.8]	14.7 [12.1; 19.8]
2.5%; 97.5% LoA, mL/min/1.73 m^2^	-15.5; 40.0	-21.0; 34.0	-25.0; 20.0	-16.5; 41.5
P_30_, %	70.0 [63.0; 75.5]^‡^	83.0 [77.0; 87.5]^‡^	89.0 [83.5; 92.0]	73.0 [66; 78.0]^‡^
Intercept	35.0 [30.5; 40.0]	37.5 [31.5; 45.5]	35.5 [31.0; 41.0]	37.0 [29.5; 46.0]
Slope	0.34 [0.26; 0.41]	0.33 [0.20; 0.43]	0.42 [0.31; 0.52]	0.30 [0.18; 0.42]
RMSE	0.127 [0.115; 0.146]	0.130 [0.117; 0.150]	0.129 [0.117; 0.147]	0.131 [0.117; 0.150]
TDI	31.18 [27.48; 34.80]	25.00 [20.00; 31.30]	18.85 [17.90; 19.86]	31.93 [26.45; 38.01]
CCC	0.405 [0.325; 0.480]	0.494 [0.403; 0.575]	0.520 [0.432; 0.600]	0.383 [0.302; 0.459]

Data are presented with 95% Confidence Intervals [95% CI]. Bias was defined as the mean difference between eGFR and mGFR. SD is the standard deviations of the difference between mGFR and eGFR. LoA: limits of agreement; P_30_: proportion of estimates 30% higher or lower than the mGFR; RMSE: root mean squared error for the regression of eGFR on mGFR; CCC: concordance correlation coefficient. Confidence intervals were calculated by a bootstrap method BCa (2,000 bootstraps).

^‡^P < 0.005 favouring LMR;

LMR equation was better than CKD-EPI, MDRD and FAS regarding accuracy P_30_ (85.5 [82.0; 89.0] vs. 70.0 [65.5; 75.0], 81.0 [77.0; 85.0] and 70.5 [65.5; 74.0] respectively, P<0.001, respectively) (Table 3). However, all equations had similar RMSE (Table 3).

In KTR with mGFR <45 mL/min/1.73 m^2^, the LMR equation was superior to CKD-EPI and FAS regarding mean bias [95%CI] (-2.0 [-3.0; -0.5] versus 4.5 [3.0; 6.0] and 7.0 [6.0; 8.5] mL/min/1.73 m^2^) and accuracy P_30_ [95%CI] (82.0 [75.0; 87.0] vs. 70.5 [63.0; 76.0], and 66.5 [59.0; 72.5], P <0.005) (Table 3). However, LMR was similar MDRD equation in mean bias (2.5 [1.0; 4.0]) and P_30_ (79.0 [72.0; 83.5]).

Results are similar whatever the method used (urinary inulin or plasmatic iohexol clearance) even if LMR performance is slightly better compared to MDRD with inulin clearance (S1–S3 Tables).

### 3.3 Agreement analysis

In the total population, the LMR equations had the best TDI [95%CI] 17.10 [16.41; 17.88] (Table 3). This indicates that 90% of eGFR showed an error ranging from −17.10 to +17.10% when compared with the mGFR. In KTR with mGFR <45 mL/min/1.73 m^2^, LMR and MDRD equations were similar concerning to TDI (Table 3).

**Fig 3:**
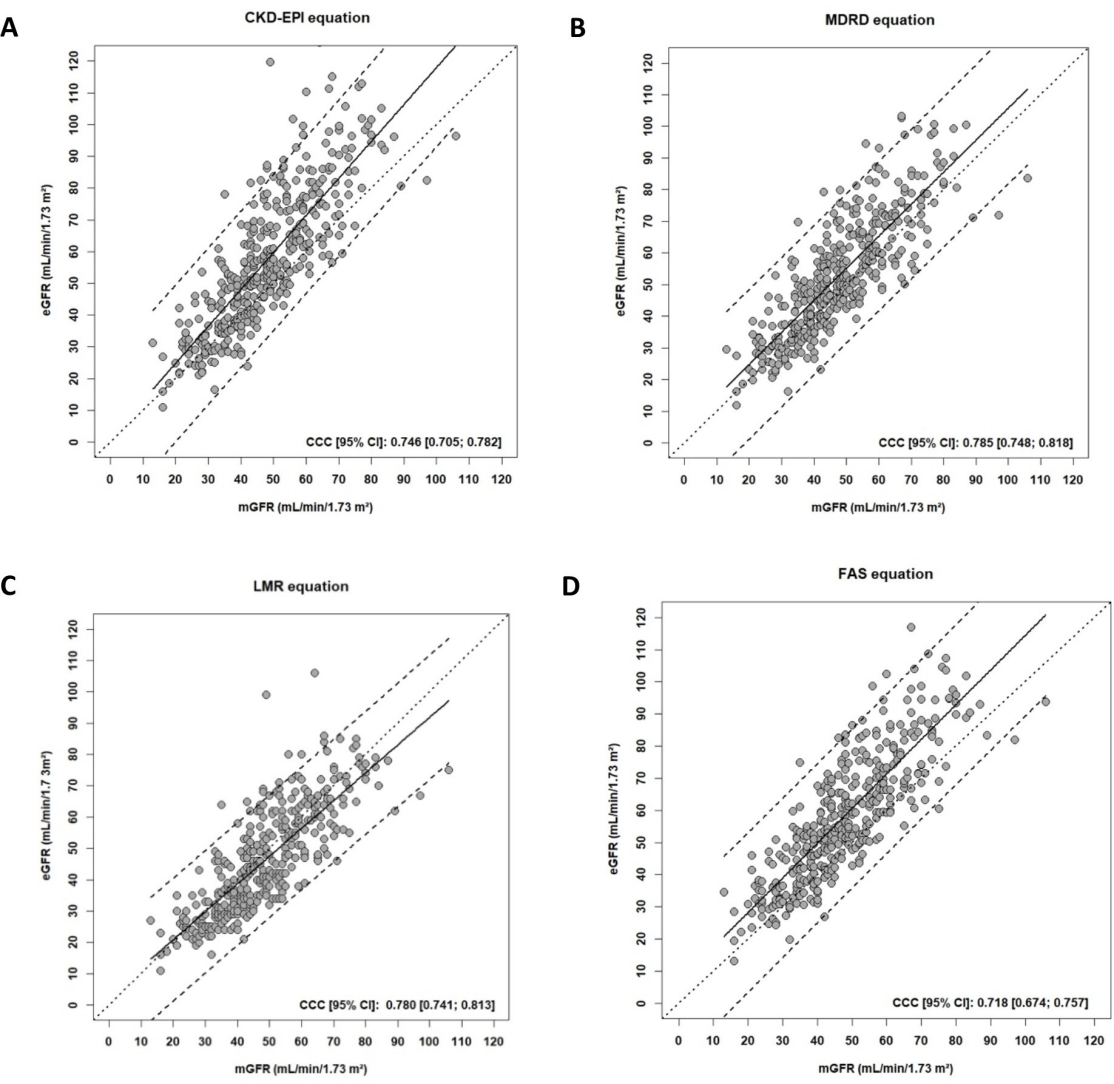
Scatterplots showing, for each equation, the estimated GFR versus the measured GFR (in mL/min/1.73 m^2^) using the different equations CKD-EPI (A), MDRD (B), LMR (C) and FAS (D). The plain lines represent the line regression. The dashed lines represent the 95% predictive confidence limits. The dotted lines represent the perfect concordance. Abbreviations: mGFR, measured glomerular filtration rate; eGFR, estimated glomerular filtration rate; CCC, Concordance Correlation Coefficient, CKD-EPI, Chronic Kidney Disease Epidemiology Collaboration; MDRD, Modification of Diet in Renal Disease Study; LMR, Lund-Malmö Revised; FAS: Full Age Spectrum; CI: confidence interval.

Concerning the CCC, all equation had poor agreement (<0.800) without statically difference between them (Fig 3) (Table 3).

### 3.4 Receiver-operator characteristic curve analysis

**Fig 4:**
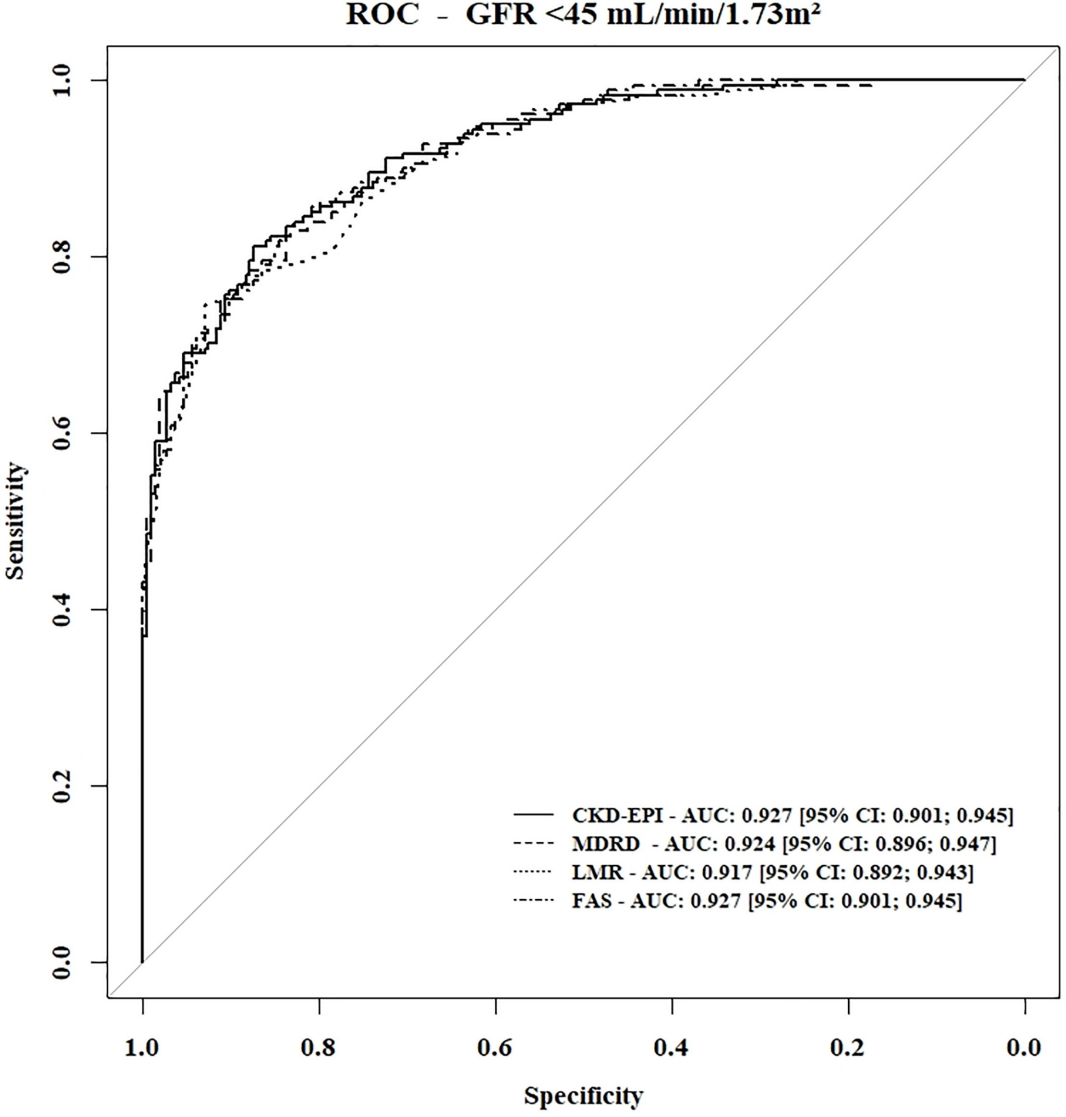
Receiver Operating Characteristic (ROC) analysis for the ability of the different GFR estimating formulas CKD-EPI, MDRD, LMR and FAS to detect mGFR< 45 mL/min/1.73 m^2^. Abbreviations: CKD-EPI, Chronic Kidney Disease–Epidemiology Collaboration; MDRD, Modification of Diet in Renal Disease Study; LMR, Lund-Malmö Revised; FAS: Full Age Spectrum; CI: confidence interval.

There was no significant difference in the AUCs of the CKD-EPI, MDRD, LMR and FAS equation (Table 4, p = 0.3) to mGFR <45 mL/min/1.73 m^2^. However, the LMR equation had the best positive likelihood ratio [95% CI]: 8.87 [5.79; 13.52] (Table 4 and Fig 4).

**Table 4:** Receiver-Operating Curve (ROC) analysis of equations estimating GFR to predict a mGFR <45mL/min/1.73m^2^ (n = 191).

	CKD-EPI equation	MDRD equation	LMR equation	FAS equation
AUC	0.927 [0.901; 0.945]	0.924 [0.896; 0.947]	0.917 [0.892; 0.943]	0.927 [0.901; 0.945]
Sensitivity	0.856 [0.803; 0.909]	0. 862 [0.810; 0.915]	0.778 [0.715; 0.841]	0.868 [0.817; 0.920]
Specificity	0.851 [0.805; 0.897]	0.820 [0.770; 0.870]	0.912 [0.876; 0.949]	0.820 [0.770; 0.870]
Positive predictive value	0.808 [0.750; 0.866]	0.778 [0.720; 0.838]	0.867 [0.812; 0.921]	0.780 [0.720; 0.840]
Negative predictive value	0.890 [0.848; 0.931]	0.890 [0.848; 0.933]	0.849 [0.804; 0.894]	0.895 [0.853; 0.936]
Positive likelihood ratio	5.70 [4.20; 7.90]	4.80 [3.61; 6.38]	8.87 [5.79; 13.52]	4.83 [3.64; 6.12]
Negative likelihood ratio	0.17 [0.11; 0.25]	0.89 [0.85; 0.93]	0.24 [0.18; 0.32]	0.16 [0.11; 0.25]

Data are presented with 95% Confidence Intervals [95% CI]. AUC: the area under a receiver curve operating characteristic. Confidence intervals were calculated by a bootstrap method (2,000 bootstraps)

## 4. Discussion

The present study found that the new LMR equation is slightly but significantly better than other equations, especially in comparison to the KDIGO recommended equation (the CKD-EPI equation).

The CKD-EPI equation is recommended for estimating GFR in adults of any age in North America, Europe, and Australia.^2^ The CKD-EPI equation was developed in 2009 in a North American and European population of 3,896 CKD (including non-CKD patients) with a wide age range (mean: 50 years) and a mean ± SD mGFR (urinary iothalamate clearance) 68.0 ± 40 mL/min/1.73 m^2^; but the proportion of KTR within the CKD-EPI internal validation datasets was only 4.0%.[2, 22] In the present study, we found a similar performance of CKD-EPI to FAS, but inferior to MDRD and LMR irrespective of the level of mGFR.

The original MDRD study equation was developed in 1999 using 1,628 CKD patients (none were KTR) and urinary iothalamate clearance for mGFR.[21] A major limitation of this equation in the general population is the underestimation of GFR in patients with normal or subnormal PCr concentration, which essentially translates to an overestimation of CKD prevalence in patients with CKD stage I-II. Zahran et al. compared the performance of 14 PCr-based equations in KTR patients and reported heterogeneous results with mean bias varying from 3.3 to 25.3 mL/min/1.73 m^2^ and P_30_ accuracy from 32.5% to 70% when mGFR was < 60 mL/min/1.73m^2^.[24] These differences can be explained by sample size, demographic characteristics, the various reference methods used for mGFR determination and non-standardization of the PCr assay in some studies. Our population of KTR patients is comparable to those of other studies in terms of GFR level as the majority of reported KTR population have an eGFR<60 mL/min/1.73 m^2^ after one year.[3, 5, 9, 10, 12, 14, 25–27]. In the present study, the mean mGFR one year after graft was close to the other KTR populations i.e. 48.0 ± 15.1 ml/min/1.73m^2^.[5, 9–11, 13] One study evaluated also the performance of MDRD in transplanted patients of various organs (53% of KTR recipients) from 5 different clinical populations and have reported a mean absolute bias of 10.6 (99.6% CI, 10.1–11.1) and a P_30_ of 78.9% (99.6% CI, 76.9%-80.8%) which is close to the results obtained in the present study.[10] In addition, several previous studies reported superior performance of the MDRD equation compared to CKD-EPI in KTR.[10, 11, 13, 27, 28] The present study found that the performance of MDRD is slightly inferior to LMR equation, with a greater difference when inulin is the reference method.

The LMR equation was developed in a Swedish Caucasian cohort including 850 individuals aged 18–95 years (median: 60 years) and using iohexol clearance plasma.[17] In recent publication, the LMR equation showed the best performance in 263 KTR aged >60 years with a mGFR <60 mL/min/1.73m^2^.[26] In the present study, the LMR equation predicted GFR more precisely and accurately than MDRD, CKD-EPI, and FAS equations in the total KTR population. Several hypothesis could be given to explain this result: first, the possible difference of GFR determination method for the development of MDRD et LMR equations (urinary iothalamate clearance and plasma iohexol clearance, respectively) and secondly the fact that LMR equation was developed with the goal of improving estimations at low mGFR levels. [17]

The FAS equation has a simple structure compared with that of the MDRD, CKD-EPI, and LMR equations; it is based on standardization of PCr: PCr/Q, where Q is the median PCr of a healthy population to account for age and gender.[18] The equation had the same performance in children, adolescents, adults, and older persons in a population of 6,870 healthy and CKD patients (none were KTR) who had a mean mGFR (iohexol, iothalamate and inulin clearance) 67.2 ±13.3 mL/min/1.73m^2^. Among KTR herein, the FAS equation was inferior to all equations probably due to the specific characteristics of the KTR population.

Finally, in the whole population and in all subgroups, none of the equations showed a P_30_ >90%, which is the KDOQI criteria of performance according to KDOQI recommendations [2] and all the equations had a CCC under 0.805, *i*.*e*. poor agreement demonstrating again that estimation of GFR with PCr based equations is inadequate and, in our opinion, mGFR using a reference method should be recommended in this specific population.

The strengths of the present study include the use of a population of KTR at one-year post-transplantation, the use of standardized assays for PCr; and the use of rigorous statistical methods for diagnostic test evaluation using continuous variables. The study has, however, several limitations. First, although not collected, the source population is known to be predominantly European [11, 25] and the results cannot be extended to other ethnic populations. Second, it was conducted in a single regional center. Third, the performance of eGFR equations in mGFR <30 or >90 mL/min/1.73 m^2^ were not specifically examined because of the small number of patients in these subgroups. Fourth, the use of PCr alone as endogenous marker (without cystatin C) has some well-known limitations in KTR.[6, 13]

## Conclusion

To our knowledge, the study is the first that compared FAS and LMR in kidney transplantation. The present evaluation of four PCr-based equations suggests that the LMR has the best mean bias and TDI in 395 kidney transplant recipients, but with no significant superiority in other agreement tool. However, performance of all the studied formulas are quite poor in renal transplant patients compared to CKD population. Caveat is required when PCr-based equations is applied in this specific population. In our opinion, renal graft function requires a reference method of GFR measurement (eg. iohexol clearance).

## Supporting information

S1 Table Estimation of the bias (mean bias estimated–measure glomerular filtration rate) with equations according of the reference method Glomerular Filtration Rate (GFR) in the whole GFR category and in different GFR categories. (DOCX)

S2 Table Accuracy P_30_ with equations according of the reference method method Glomerular Filtration Rate (GFR) in the whole GFR category and in different GFR categories. (DOCX)

S3 Table The Total Deviation Index estimated (TDI) of the reference method Glomerular Filtration Rate (GFR) in the whole sample and in different GFR categories. (DOC)

## Abbreviations

AUC: area under the receiver-operating curve
CCC: Concordance Correlation Coefficient
CI: confidence interval
CKD: chronic kidney disease
CKD-EPI: Chronic Kidney Disease–Epidemiology Collaboration equation
eGFR: estimated glomerular filtration rate
FAS: Full Age Spectrum
GFR: glomerular filtration rate
HPLC: High Performance Liquid Chromatography
IDMS: isotope-dilution mass spectrometry-calibrated
IQR: interquartile range
KTR: kidney transplantation recipients
LCMS: liquid chromatography mass spectrometry
LMR: Lund-Malmö Revised
MDRD: Modification of Diet in Renal Disease Study equation
mGFR: measured glomerular filtration rate
PCr: plasma creatinine
PLR: likelihood ratio for a positive result
RMSE: root mean square error
ROC: receiver-operating curve
