# Supplementary material for: Comparison of creatinine-based equations for estimating glomerular filtration rate in deceased donor renal transplant recipients
Source: PLoS One. 2020 Apr 28;15(4):e0231873. doi: 10.1371/journal.pone.0231873 (PMC7188287; doi:10.1371/journal.pone.0231873)
Supplement: S1 Table — (DOCX) [file pone.0231873.s001.docx]

**Supplementary Table 1- Estimation of the bias (mean bias estimated – measure glomerular filtration rate ) with equations according of the reference method glomerular filtration rate (GFR) in the whole GFR category and in different GFR categories.**

| **GFR category** | **Group** | **BIAS (95% CI)** | | | |
| --- | --- | --- | --- | --- | --- |
|  |  | **CKD-EPI** | **MDRD** | **LMR** | **FAS** |
| Whole GFR category | Iohexol clearance | 7.5 (6.0; 9.0)^‡^ | 3.0 (2.0; 4.0) | -3.5 (-5.0; -2.5) | 9.0 (8.0; 10.0)^‡^ |
|  | Inulin clearance | 14.5 (11.0; 18.0)^‡^ | 10.5 (7.0; 14.0)^‡^ | 2.5 (-1.0; 6.0) | 16.0 (12.0; 19.0)^‡^ |
| GFR <45mL/min/1.73 m^2^ | Iohexol clearance | 3.0 (1.5; 5.0) | 1.0 (-1.0; 2.0) | -3.0 (-4.0; -1.5) | 5.0 (3.0; 7.0) |
|  | Inulin clearance | 11.0 (7.5; 14.0)^‡^ | 8.0 (5.0; 11.0)^‡^ | 2.0 (0.0; 5.0) | 13.0 (10.5; 16.0)^‡^ |
| GFR ≥45 mL/min/1.73 m^2^ | Iohexol clearance | 11.0 (9.0; 12.5)^‡^ | 5.0 (3.0; 6.0) | -4.0 (-5.5; -2.5) | 11.0 (9.0; 12.5)^‡^ |
|  | Inulin clearance | 18.5 (12.5; 24.0)^‡^ | 13.0 (6.0; 19.5)^‡^ | 2.5 (-2.0; 7.0) | 18.5 (11.5; 25.5)^‡^ |

GFR: glomerular filtration rate, ^‡^P<0.005 favoring LMR
