# Supplementary material for: Comparison of creatinine-based equations for estimating glomerular filtration rate in deceased donor renal transplant recipients
Source: PLoS One. 2020 Apr 28;15(4):e0231873. doi: 10.1371/journal.pone.0231873 (PMC7188287; doi:10.1371/journal.pone.0231873)
Supplement: S2 Table — (DOCX) [file pone.0231873.s002.docx]

**Supplementary Table 2 - Accuracy P_30_ with equations according of the reference method method glomerular filtration rate (GFR) in the whole GFR category and in different GFR categories.**

| **GFR category** | **Group** | **P_30_ (95% CI)** | | | |
| --- | --- | --- | --- | --- | --- |
|  |  | **CKD-EPI** | **MDRD** | **LMR** | **FAS** |
| Whole GFR category | Iohexol clearance | 74.5 (72.0; 77.0)^‡^ | 85.5 (83.0; 87.5) | 87.0 (85.0; 88.5) | 74.5 (71.5; 76.0)^‡^ |
|  | Inulin clearance | 54.0 (48.5; 59.0)^‡^ | 64.0 (58.0; 69.0)^‡^ | 81.0 (76.0; 84.5) | 53.0 (48.0; 58.0)^‡^ |
| GFR <45mL/min/1.73 m^2^ | Iohexol clearance | 75.0 (71.0; 78.5)^‡^ | 82.0 (78.5; 85.0) | 83.0 (79.5; 86.0) | 71.5 (67.0; 74.5)^‡^ |
|  | Inulin clearance | 51.0 (42.5; 58.0)^‡^ | 63.5 (55.0; 69.5)^‡^ | 83.0 (76.0; 88.0) | 44.0 (36.0; 51.0)^‡^ |
| GFR ≥45 mL/min/1.73 m^2^ | Iohexol clearance | 74.0 (70.5; 77.0)^‡^ | 88.0 (85.0; 90.5) | 91.0 (88.5; 93.0) | 76.0 (73.0; 79.5)^‡^ |
|  | Inulin clearance | 54.0 (46.0; 61.0)^‡^ | 61.5 (53.0; 68.5)^‡^ | 79.5 (72.0; 84.5) | 59.0 (50.5; 66.0)^‡^ |

GFR: glomerular filtration rate, ^‡^P<0.005 favoring LMR
