# Supplementary material for: Comparison of creatinine-based equations for estimating glomerular filtration rate in deceased donor renal transplant recipients
Source: PLoS One. 2020 Apr 28;15(4):e0231873. doi: 10.1371/journal.pone.0231873 (PMC7188287; doi:10.1371/journal.pone.0231873)
Supplement: S3 Table — (DOC) [file pone.0231873.s003.doc]

**Supplementary Table 3- The Total Deviation Index estimated (TDI) of the reference method glomerular filtration rate (GFR)** in the whole sample and in different GFR categories.

| **GFR category** | **Group** | **TDI (95% CI)** | | | |
| --- | --- | --- | --- | --- | --- |
|  |  | **CKD-EPI** | **MDRD** | **LMR** | **FAS** |
| Whole GFR category | Iohexol clearance | 23.03 (22.00; 24.09) | 17.28 (16.46; 18.08) | 15.85 (15.25; 16.43) | 22.62 (21.70; 23.52) |
|  | Inulin clearance | 35.00 (32.00; 38.35) | 32.40 (27.18; 37.62) | 20.45 (17.90; 23.08) | 38.81 (33.28; 44.82) |
| GFR <45mL/min/1.73 m^2^ | Iohexol clearance | 17.26 (16.18; 18.36) | 14.64 (13.74; 15.60) | 13.40 (12.72; 14.00) | 18.14 (17.14; 19.11) |
|  | Inulin clearance | 24.94 (21.73; 28.00) | 20.70 (17.76; 23.41) | 13.73 (11.60; 16.15) | 26.50 (24.21; 28.93) |
| GFR ≥45 mL/min/1.73 m^2^ | Iohexol clearance | 26.50 (25.50; 28.50) | 19.20 (18.00; 20.41) | 17.61 (16.70; 18.51) | 25.88 (24.54; 27.22) |
|  | Inulin clearance | 44.00 (38.95; 49.17) | 42.22 (33.70; 50.74) | 26.00 (22.20; 30.03) | 49.80 (40.86; 58.43) |

|  |
| --- |
